# Tetrandrine Attenuates Podocyte Injury by Inhibiting TRPC6-Mediated RhoA/ROCK1 Pathway

**DOI:** 10.1155/2022/7534181

**Published:** 2022-09-30

**Authors:** Lichan Mao, Yin Ding, Dongrong Yu, Jiazhen Yin, Jin Yu

**Affiliations:** Department of Nephrology, Hangzhou TCM Hospital Affiliated to Zhejiang Chinese Medical University, Hangzhou 310007, China

## Abstract

Tetrandrine (Tet), a compound found in a traditional Chinese medicine, presents the protective effect for kidney function. Our study is aimed at clarifying the efficacy and underlying mechanism of Tet on podocyte injury. In this study, podocyte injury was induced in rats with adriamycin (ADR), and MPC5 podocytes were constructed with TRPC6 overexpression. We found that Tet treatment reduced the levels of proteinuria, serum creatinine, and blood urea nitrogen and increased plasma albumin levels in ADR-induced rats. Tet reduced intracellular Ca^2+^ influx and apoptosis in MPC5 podocytes overexpressing TRPC6. Tet downregulated the expression of renal TRPC6, RhoA, and ROCK1 and upregulated the expression of synaptopodin; meanwhile, it reduced calcineurin activity *in vivo* and *in vitro*. In conclusion, Tet protects against podocyte by affecting TRPC6 and its downstream RhoA/ROCK1 signaling pathway.

## 1. Introduction

Podocyte function plays an important role in the pathogenesis of kidney diseases. Podocyte injury can cause the increase of extracellular matrix, basement membrane thickening, fibrosis factor upregulation, and renal failure [1–3]. The protection of podocyte morphology and function is crucial for maintaining healthy kidney function.

Fangji Huangqi decoction (FHD), a classic prescription of traditional Chinese medicine used to treat nephrotic syndrome, has proved its effect on protecting podocyte [4–6]. Tetrandrine (Tet) is a major active component of Fang Ji (*Stephania tetrandra*) in FHD [7, 8], though the precise role and mechanism of Tet in renoprotection remain unclear. Tet is a nonselective Ca^2+^ antagonist which blocks L-type Ca^2+^ channels, thereby lowering blood pressure [9, 10]. Tet also has an antifibrotic effect on the kidney due to its ability to upregulate expression of matrix metallopeptidase 13 that activates the transforming growth factor *β*/Smads signaling pathway, thereby reducing downstream connective tissue growth factor expression [10]. Podocyte injury is the classical pathological process in multiple glomerular diseases, resulting in kidney dysfunction [11]. Based on the background mentioned above, we speculated that Tet may protect against podocyte injury in kidney diseases.

Transient receptor potential cation channel 6 (TRPC6) is a prominent component of the podocyte slit diaphragm, where it interacts with podocin and nephrin to maintain normal podocyte morphology and function [12–14]. TRPC6 overexpression is found in both acquired and genetic nephropathies and is strongly associated with proteinuria and impaired renal function [12]. TRPC6 contributes greatly toward Ca^2+^ influx into podocytes and further promotes the activitity of RhoA [15–18]. The abnormal activation of RhoA, and its downstream effector Rho-associated coiled coil-containing protein kinase1 (ROCK1) are closely related to the disorder of actin cytoskeleton, contraction, and apoptosis of podocytes [16, 19–22]. Therefore, inhibition of TRPC6 expression may be an important way to protect podocyte. Meanwhile, we tend to explore whether Tet alleviates podocyte injury via regulating TRPC6.

The current study determines the *in vitro* and *in vivo* therapeutic effects of Tet on podocyte injury through evaluating proteinuria, blood kidney function indexes, podocyte morphology, and intracellular Ca^2+^ influx. Simultaneously, the molecular mechanism of Tet against podocyte injury involving in the TRPC6-mediated RhoA/ROCK1 signaling pathway was revealed. A flow chart of the complete experimental design has been presented in Supplementary Figure 1.

## 2. Materials and Methods

### 2.1. Animal Grouping and Treatment

Forty-eight eight-week old Sprague-Dawley (SD) rats (male : female = 1 : 1) weighing about 200 g were purchased from Changzhou Cavens Experimental Animal Co., Ltd. Rats were randomly divided into the following six groups (*n* = 8 each group): control group (normal), ADRIA group (model), Tet-low dose (Tet-LD) group, Tet-medium dose (Tet-MD) group, Tet-high dose (Tet-HD) group, and cyclosporine A (CsA) group. In order to obtain an ideal nephropathy rat model in a short period of time, we used unilateral nephrectomy combined with ADR induction to establish a nephropathy model [23, 24]. The left kidney of each rat was removed after intraperitoneal injection of 50 mg/kg ketamine for full anesthesia [25]. On the first and fourteenth day after the operation, all SD rats in the ADRIA, Tet-LD, Tet-MD, Tet-HD, and CsA groups were injected with adriamycin (ADR) through the tail vein at the dosage of 4 mg/kg body weight [26]. Rats in the control group were injected with the same volume of normal saline. After modeling, rats in Tet-LD, Tet-MD, Tet-HD, and CsA groups were given 4 mg/kg/d Tet (Sigma-Aldrich, USA), 8 mg/kg/d Tet, 16 mg/kg/d Tet, and 30 mg/kg/d CsA (Sigma-Aldrich) by gavage, respectively. The dosages of Tet and CsA used in this study were based on our previous report [27]. Rats in the control and ADRIA groups received the same volume of distilled water. Animal experiments were approved by the Institutional Animal Care and Use Committee of the Hangzhou TCM Hospital Affiliated to Zhejiang Chinese Medical University (no. 20210927-25).

### 2.2. Detection of Urine Protein, Serum Creatinine, Plasma Albumin, and Blood Urea Nitrogen

Blood samples and 24-hour urine were collected from rats after treatment for 12 weeks. Blood biochemical indexes (serum creatinine, plasma albumin, and blood urea nitrogen) and 24-hour urine protein were quantified by automatic biochemical analyzer.

### 2.3. Identification of Podocyte Morphology

Identification of podocyte morphology was performed as previously described [25]. Briefly, kidney tissues were collected from rats after treatment for 12 weeks. Tissues were fixed with 3% glutaraldehyde and 4% paraformaldehyde in 0.1 mol/L phosphate buffer. After fixation, tissues were dehydrated with gradient ethanol (50%, 70%, 95%, and 100%) and finally embedded in Durcupan resin. Podocyte morphology in kidney tissues was determined under a transmission electron microscope (JEOL, USA).

### 2.4. Cell Transfection and Treatment

In order to construct a TRPC6 overexpression lentiviral plasmid, the full-length coding sequence region of the mouse TRPC6 gene was cloned into the EcoRI/BamHI restriction sites of pcDH-GFP-PURO vector using gene synthesis (Shanghai GeneChem. Co. Ltd., China) as previously described [28]. TRPC6 overexpression plasmid was then packaged into lentivirus (lev-TRPC6) and then transfected into MPC5 podocytes. Blank and lev-negative control (lev-NC) transfecting into MPC5 podocytes were used as the control. Subsequently, MPC5 podocytes transfected with lev-TRPC6 were treated with 10 *μ*M U73122 (an inhibitor of TRPC6 channel opening) [28] for 10 min, 2 *μ*M CsA for 48 h, and 40 *μ*M Tet for 48 h. Cells were cultured at 37°C with 5% CO_2_. The drug concentrations of Tet and CsA treating MPC5 podocytes depended on the CCK-8 assay in our previous study [27].

### 2.5. qRT-PCR

MPC5 podocytes and kidney tissues were collected from rats, and RNA was extracted with TRIzol (Qiagen) as previously described [28]. TRPC6, RhoA, ROCK1, and synaptopodin were detected using previously reported primers [28].

### 2.6. Western Blot Analysis

Total protein was extracted from MPC5 podocytes and kidney tissues of rats. For sufficient cell lysis, RIPA lysis buffer (Beyotime, China) was added, and then, the supernatant was extracted after centrifugation at 12,000 rpm for 10 min. Protein samples were separated with 10% SDS-PAGE and transferred onto PVDF membranes. Membranes were blocked with skim milk powder and then incubated with primary antibodies to anti-TRPC6 (1 : 1,000; Santa Cruz, USA), RhoA (1 : 1,000; Proteintech, Wuhan, China), ROCK1 (1 : 3,000; Proteintech), synaptopodin (1 : 1,000; Santa Cruz), and GAPDH (1 : 10,000; Abcam, UK). Membranes were then incubated with donkey anti-mouse IgG (H+L; Jackson ImmunoResearch Laboratories, USA) secondary antibody. ECL system (Sharebio, China) was used for band detection and protein bands were analyzed by the Tanon Image software (Tanon, China).

### 2.7. Intracellular Ca^2+^ Assay

Ca^2+^ influx assay was performed as previously described [29]. To detect levels of intracellular Ca^2+^, MPC5 podocytes were incubated with 10 *μ*M Fluo-3AM (Donjindo Laboratories, Japan) at 37°C for 30 min, washed with PBS three times, and incubated with 10 *μ*M Fluo-3AM for another 10 min before fluorescence detection. Ca^2+^-related apoptosis was assessed by confocal imaging and analyzed by Zeiss confocal imaging system (Zeiss, USA).

### 2.8. Calcineurin (CaN) Activity Assay

CaN activity was measured in MPC5 podocytes and kidney tissues using a CaN activity kit (Jiancheng Biotech, Nanjing, China) following the manufacturer's protocol.

### 2.9. Flow Cytometry

MPC5 podocytes were mixed with 300 *μ*L binding buffer and then were labeled with AnnexinV-FITC/PI (BD Biosciences, USA). Flow cytometry (BD Biosciences) was used for analyzing the apoptosis rate as previously described [28].

### 2.10. Statistical Analysis

All data are shown as mean ± standard deviation. SPSS and GraphPad Prism 6 were used for statistical analyses by one-way analysis of variance. The threshold of significant difference was *P* < 0.05 for all tests.

## 3. Results

### 3.1. Tetrandrine Recovers Renal Function of Rats with Nephropathy

**Figure 1:**
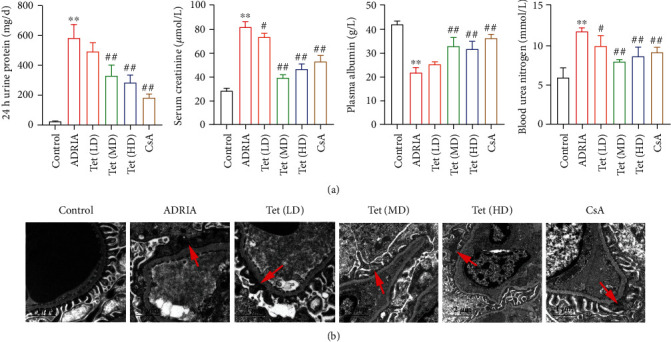
Tetrandrine (Tet) recovers the renal dysfunction and podocyte injury in adriamycin- (ADR-) induced nephropathy rats. (a) The levels of 24-hour urine protein and renal function indexes (serum creatinine, plasma albumin, and urea nitrogen) in rats. ^∗∗^*P* < 0.01 versus the control group. ^#^*P* < 0.05 and ^##^*P* < 0.01 versus the ADRIA group. (b) Process fusion (red arrows) of podocytes was identified via electron microscopy (scale bar = 1 *μ*m). Nephropathy rats were induced by adriamycin (ADRIA) and then treated with low-dose (LD), medium-dose (MD), high-dose (HD) Tet, or cyclosporine A (CsA, a positive drug).

A nephropathy rat model (ADRIA) was established by ADR induction, and model rats were treated with different doses of Tet. Proteinuria, serum creatinine, plasma albumin, and blood urea nitrogen are essential indicators of podocyte injury. Model rats showed impaired renal function, with the increased urine protein, serum creatinine, and blood urea nitrogen, as well as the decreased plasma albumin as compard with control rats (*P* < 0.01) (Figure 1(a)). After treatment with medium- and high-dose Tet, or CsA (a positive drug), ADRIA rats presented the significantly reduced levels of urine protein, serum creatinine, and blood urea nitrogen and the increased plasma albumin level (*P* < 0.01) (Figure 1(a)).

### 3.2. Tetrandrine Ameliorates Podocyte Injury in Rats with Nephropathy

To investigate whether Tet can ameliorate podocyte injury, we detected the ultrastructure of podocytes isolated from rats by electron microscopy. Extensive process fusion of podocytes (80–100%) was detected in kidney tissues of ADRIA rats group when compared to that in control rats. Process fusion was greatly reduced in podocytes extracted from ADRIA rats treated with Tet or CsA. High-dose Tet or CsA treatment showed more obvious effect (30–40%), followed by the medium-dose group (50–60%) and then the low-dose group (70–80%) (Figure 1(b)).

### 3.3. Tetrandrine Downregulates the Expression of Renal TRPC6, ROCK1, and RhoA and Upregulates the Level of Synaptopodin in ADR-Induced Nephropathy Rats

**Figure 2:**
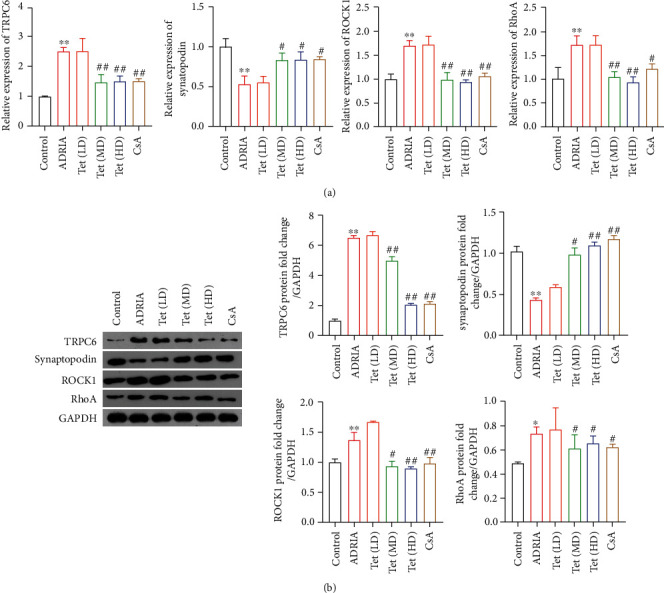
Tetrandrine (Tet) inhibits TRPC6 expression and RhoA/ROCK1 pathway *in vivo*. (a) The mRNA expression of TRPC6, synaptopodin, RhoA, and ROCK1 in kidney tissues of rats was measured by RT-qPCR. (b) The protein expression of TRPC6, synaptopodin, RhoA, and ROCK1 in kidney tissues of rats was detected by western blotting. Nephropathy rats were induced by adriamycin (ADRIA) and then treated with low-dose (LD), medium-dose (MD), and high-dose (HD) Tet, or cyclosporine A (CsA, a positive drug). ^∗^*P* < 0.05 and ^∗∗^*P* < 0.01 versus the control group. ^#^*P* < 0.05 and ^##^*P* < 0.01 versus the ADRIA group.

TRPC6, a transient receptor potential channel, is expressed in podocytes and regulates podocyte injury [30]. The mRNA and protein expressions of TRPC6 were significantly increased in ADRIA rats when compared to that in control rats (*P* < 0.01) (Figures 2(a) and 2(b)). Treatment with Tet (medium or high dose) or CsA reduced expression of TRPC6 in ADRIA rats (*P* < 0.01) (Figures 2(a) and 2(b)). In addition, synaptopodin, a mature podocyte marker, is closely related with dedifferentiated and dysregulated podocyte phenotype and limits TRPC6 podocyte surface expression [31]. ADRIA rats showed a marked decrease in the mRNA and protein expression of synaptopodin compared to control rats (*P* < 0.01) (Figures 2(a) and 2(b)). The levels of synaptopodin was increased in ADRIA rats by treatment with medium or high doses of Tet or CsA (*P* < 0.05) (Figures 2(a) and 2(b)). Furthermore, it has been reported that TRPC6 can activate RhoA/ROCK1 signaling, thereby inducing podocyte injury [28]. As shown in Figures 2(a) and 2(b), RhoA and ROCK1 expressions were elevated in ADRIA rats as compared to that in control rats, whereas treatment with Tet (medium or high dose) or CsA reduced the levels of RhoA and ROCK1 in ADRIA rats (*P* < 0.05).

### 3.4. Tetrandrine Mitigates the Podocyte Injury and the Intracellular Ca^2+^ Influx via the Blockage of TRPC6-Mediated RhoA/ROCK1 Pathway

**Figure 3:**
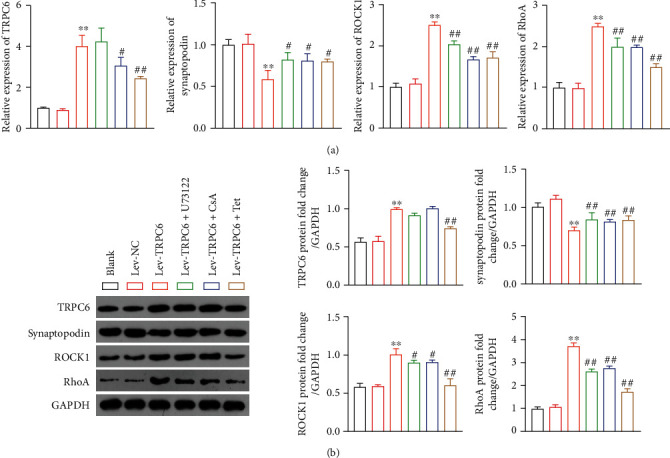
Tetrandrine (Tet) supresses the TRPC6-mediated RhoA/ROCK1 pathway in podocytes. (a) The mRNA expression of TRPC6, synaptopodin, RhoA, and ROCK1 in MPC5 podocytes was measured by RT-qPCR. (b) The protein expression of TRPC6, synaptopodin, RhoA, and ROCK1 in MPC5 podocytes was determined by western blotting. MPC5 podocytes were transfected with lev-NC or lev-TRPC6 and then treated with U73122 (an inhibitor of TRPC6 channel opening), CsA, or Tet. ^∗∗^*P* < 0.01 versus the lev-NC group. ^#^*P* < 0.05 and ^##^*P* < 0.01 versus the lev-TRPC6 group.

To further decipher whether Tet exerts the protective effect on podocyte injury, TRPC6 was overexpressed in MPC5 podocytes that were then treated with U73122 (an inhibitor of TRPC6 channel opening), CsA, or Tet. RT-qPCR and western blotting showed that TRPC6 overexpression increased the expression of TRPC6, RhoA, and ROCK1 and decreased the synaptopodin level in MPC5 podocytes in comparison to the lev-NC (*P* < 0.01) (Figures 3(a) and 3(b)). However, the treatment of U73122, CsA, or Tet downregulated the expression of TRPC6, RhoA, and ROCK1 and upregulated the synaptopodin level in MPC5 podocytes transfected with lev-TRPC6 (*P* < 0.05) (Figures 3(a) and 3(b)).

**Figure 4:**
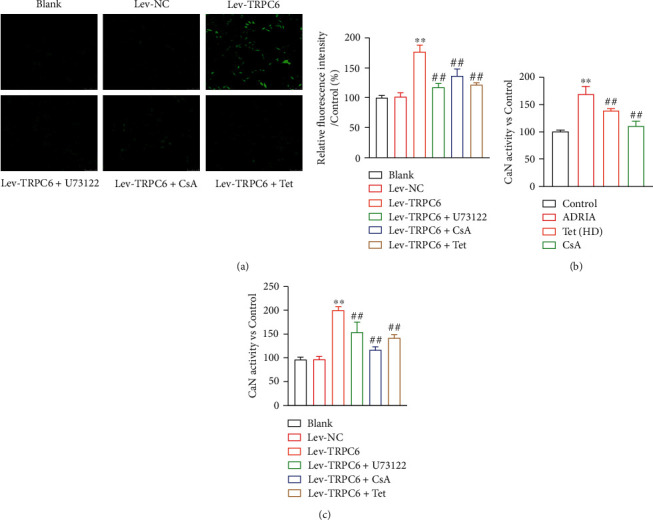
Tetrandrine (Tet) reduced intracellular Ca^2+^ influx and calcineurin (CaN) activity *in vitro* and *in vivo*. (a) The intracellular Ca^2+^ influx in MPC5 podocytes was identified by fluorescence detection (scale bar = 100 *μ*m). ^∗∗^*P* < 0.01 versus the lev-NC group. ^##^*P* < 0.01 versus the lev-TRPC6 group. (b, c) The CaN activities in rats and in MPC5 podocytes. ^∗∗^*P* < 0.01 versus the control or lev-NC group. ^##^*P* < 0.01 versus the ADRIA or lev-TRPC6 group. Nephropathy rats were induced by adriamycin (ADRIA) and then treated with high-dose Tet (16 mg/kg/d) or cyclosporine A (CsA, a positive drug). MPC5 podocytes were transfected with lev-NC or lev-TRPC6 and then treated with U73122 (an inhibitor of TRPC6 channel opening), CsA, or Tet.

In addition, Ca^2+^ signaling in podocytes reportedly results in proteinuria and podocyte injury [32]. Intracellular Ca^2+^ influx in MPC5 podocytes with TRPC6 overexpression was significantly higher than that in lev-NC group (*P* < 0.01) (Figure 4(a)). The administration of U73122, CsA, and Tet significantly inhibited the intracellular Ca^2+^ influx in MPC5 podocytes transfected with lev-TRPC6 (*P* < 0.01) (Figure 4(a)). Moreover, we found that CaN activity was significantly higher in ADRIA rats than that in control rats, which was inhibited by high-dose Tet (16 mg/kg/d) or CsA treatment (*P* < 0.01) (Figure 4(b)). Meanwhile, CaN activity was markedly higher in MPC5 podocytes with TRPC6 overexpression than that in the lev-NC group, which was reduced by treatment with U73122, CsA, or Tet (*P* < 0.01) (Figure 4(c)).

### 3.5. Tetrandrine Suppresses the Podocyte Apopotosis via the Blockage of TRPC6-Mediated RhoA/ROCK1 Pathway

**Figure 5:**
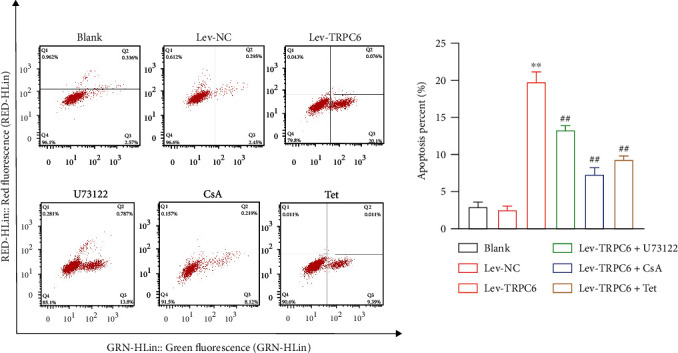
Tetrandrine (Tet) represses apoptosis of podocytes via targeting TRPC6. The apoptosis percent of MPC5 podocytes was detected by flow cytometry analysis. ^∗∗^*P* < 0.01 versus the lev-NC group. ^##^*P* < 0.01 versus the lev-TRPC6 group. MPC5 podocytes were transfected with lev-NC or lev-TRPC6 and then treated with U73122 (an inhibitor of TRPC6 channel opening), CsA, or Tet.

Podocyte apoptosis is a critical mechanism, resulting in proteinuria in various chronic kidney diseases [33]. Flow cytometry showed that TRPC6 overexpression increased the apoptotic proportion of MPC5 podocytes when compared with lev-NC (*P* < 0.01) (Figure 5). U73122, CsA, or Tet treatment remarkablely reduced the TRPC6 overexpression-mediated increase of podocyte apoptosis (*P* < 0.01) (Figure 5).

## 4. Discussion

Tetrandrine (Tet) is a bisbenzylisoquinoline alkaloid from Chinese medicine herb *Stephania tetrandra*, possessing promising anticancer, anti-inflammatory, and antiproteinuric properties [27, 34, 35]. Podocyte injury is a major pathological feature of proteinuric kidney disease, and the identification of potential therapeutic targets for alleviating podocyte injury has clinical importance [36]. Here, a nephropathy rat model (ADRIA) was established by unilateral nephrectomy combined with ADR induction to determine the therapeutic effects of Tet on podocyte injury. Proteinuria, serum creatinine, and blood urea nitrogen elevation and plasma albumin reduction are the main clinical signature of podecyte injury [37], which were exhibited in ADRIA rats. Our study found that Tet treatment reduced the levels of unrine protein, serum creatinine, and blood urea nitrogen and increased the plasma albumin level in ADRIA rats, confirming the therapeutic efficacy of Tet on podocyte injury. Moreover, transmission electron microscopy showed that podocyte fusion was increased in kidney tissues of ADRIA rats, which was mitigated by Tet treatment.

TRPC6 has become an important target for the treatment of podocyte-associated nephropathy [12]. Increased expression of TRPC6 leads to podocyte injury [38]. Our study showed that Tet has a protective effect on podocyte injury via inhibiting TRPC6 overexpression. ADR-induced nephropathy rats presented the increased expression of TRPC6 and extensive process fusion of podocytes, accompanied by massive proteinuria and renal dysfunction. In addition, podocytes overexpressing TRPC6 had increased intracellular concentrations of Ca^2+^ and apoptosis. These malignant characteristics of podocyte injury in ADRIA rats were attenuated by Tet treatment.

Additionally, we found the increased expression of RhoA/ROCK1 and CaN activity in ADRIA rats and TRPC6 overexpressed podocytes were reversed by Tet. Activation of RhoA is Ca^2+^ dependent and inhibited by the treatment of BAPTA-AM, a kind of Ca^2+^ chelator [20]. TRPC6 overexpression increases Ca^2+^ influx and further influences RhoA activation, thus activating the RhoA/ROCK1 signal pathway. The RhoA/ROCK1 pathway is closely related to recombination of microtubules and actin filaments in podocytes. Abnormal activation of RhoA/ROCK1 may induce the disorder of actin cytoskeleton, contraction, and apoptosis of podocytes [16, 19–22]. CaN is another effctor activated by Ca^2+^ influx induced by TRPC6 [12]. It is a Ca^2+^-dependent phosphatase known to lead to dephosphorylation of synotopodin and activating nuclear factor of activated T cells (NFAT), which is closely related to podocyte injury and kidney disease [39]. Synaptopodin, as an actin-binding protein, plays a major role in maintaining the cytoskeleton of podocytes [40, 41].

CsA, a well-known calcineurin inhibitor, is widely used to treat nephrotic syndrome. Previous studies suggest that the renal protective effect of CsA is related to immune regulation by the activation of NFAT. Recent studies suggest that CsA also acts directly on podocytes by inhibiting the dephosphorylation and degradation of synaptopodin and reducing CaN activity [31, 41]. In our findings, Tet inhibited the increased CaN activity triggered by ADR *in vivo* and by lev-TRPC6 *in vitro*.

On the basis of the above data, we speculate that Tet might protect podocytes by affecting the expression of the TRPC6-mediated RhoA/ROCK1 signaling pathway. Our study suggests that Tet may improve therapeutic effects for podocyte injury.

## 5. Conclusion

Our study confirmed that Tet has a protective effect on podocytes. Differing from CsA, Tet might protect podocytes mainly by affecting the TRPC6-mediated RhoA/ROCK1 signaling pathway. Further studies should be conducted to determine whether these drugs work synergistically or have any side effects and recurrence rate.

## Data Availability

The data that support the findings of this study are available from the corresponding author upon reasonable request.

## Supplementary Materials

Supplementary Materials Figures S1: a flow chart of complete experimental design for this study.
