# Supplementary figures and images for: Tetrandrine Attenuates Podocyte Injury by Inhibiting TRPC6-Mediated RhoA/ROCK1 Pathway
Source: Anal Cell Pathol (Amst). 2022 Sep 30;2022:7534181. doi: 10.1155/2022/7534181 (PMC9553700; doi:10.1155/2022/7534181)

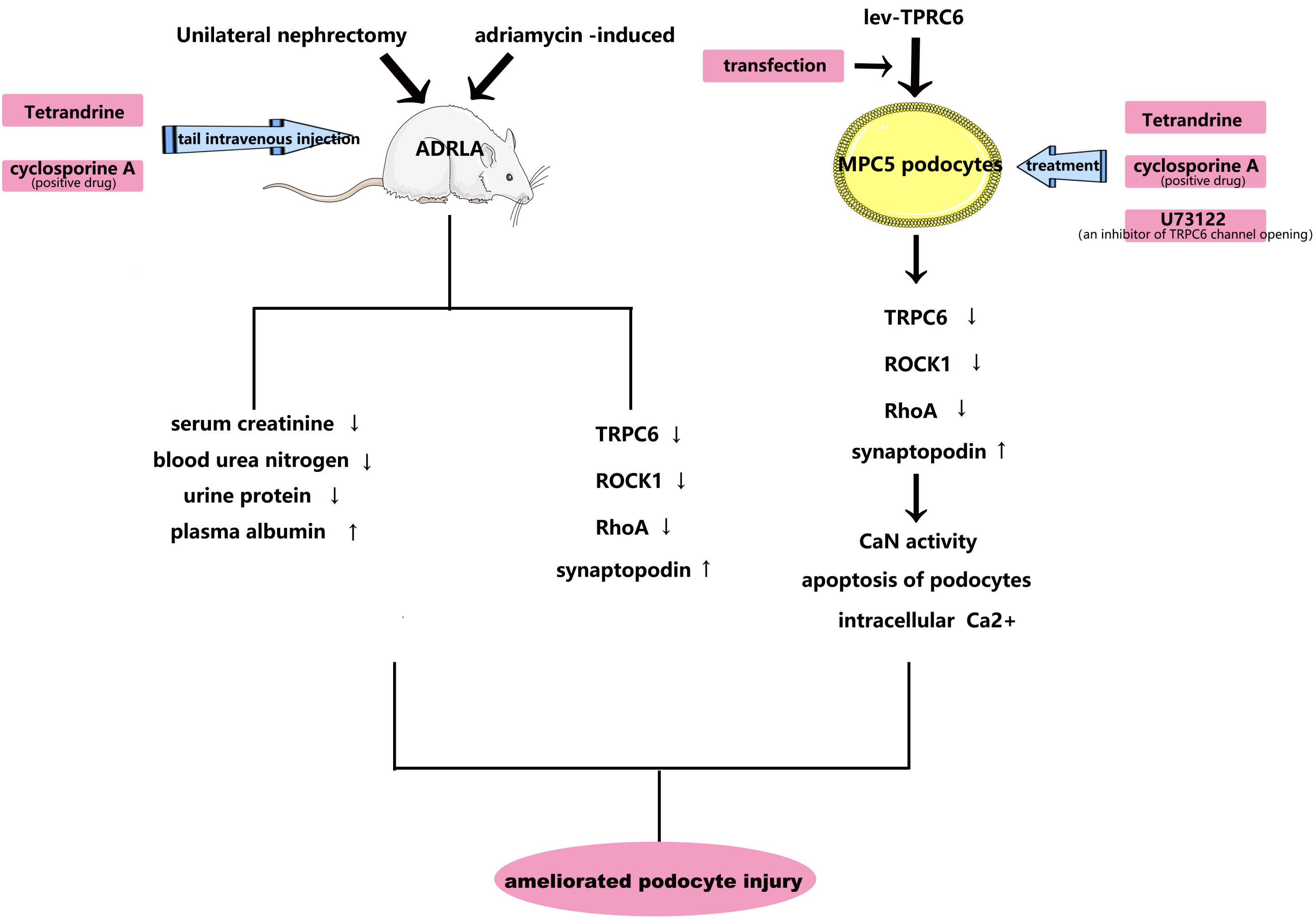

Supplement: Supplementary Materials — Figures S1: a flow chart of complete experimental design for this study. [file 7534181.f1.pdf]
